# Simulation and Analysis of a Metamaterial Sensor Based on a Microring Resonator

**DOI:** 10.3390/s110605886

**Published:** 2011-05-31

**Authors:** Ming Huang, Jingjing Yang, Sun Jun, Shujuan Mu, Yaozhong Lan

**Affiliations:** 1 School of Information Science and Engineering, Yunnan University, Kunming 650091, China; E-Mails: shujuanmu@yahoo.cn (S.M.); yaozhonglan_ynu@yahoo.cn (Y.L.); 2 Faculty of Materials and Metallurgical Engineering, Kunming University of Science and Technology, Kunming 650093, China; E-Mail: sunjun_kmustt@yahoo.cn

**Keywords:** sensor, metamaterials, microring

## Abstract

Metamaterials are artificial media structured on a size scale smaller than the wavelength of external stimuli, that may provide novel tools to significantly enhance the sensitivity and resolution of the sensors. In this paper, we derive the dispersion relation of hollow cylindrical dielectric waveguide, and compute the resonant frequencies and Q factors of the corresponding Whispering-Gallery-Modes (WGM). A metamaterial sensor based on microring resonator operating in WGM is proposed, and the resonance intensity spectrum curves in the frequency range from 185 to 212 THz were studied under different sensing conditions. Full-wave simulations, considering the frequency shift sensitivity influenced by the change of core media permittivity, the thickness and permittivity of the adsorbed substance, prove that the sensitivity of the metamaterial sensor is more than 7 times that of the traditional microring resonator sensor, and the metamaterial layer loaded in the inner side of the microring doesn’t affect the high Q performance of the microring resonator.

## 1. Introduction

Metamaterials, which are artificial materials whose permittivity and/or permeability can be designed to continuously change from negative to positive values, have attracted considerable attention for their intriguing electromagnetic properties. Many novel applications have been proposed based on metamaterials, such as perfect lenses, cloaks, concentrators, directive antennae, superscatterers, superabsorbers, transparent devices and so on [1–5]. Recently, a great deal of interest has been devoted to the potential sensing applications of metamaterials. For example, He *et al.* [6], studied the resonant modes of a 2D subwavelength open resonator, and showed it was suitable for biosensing. Jakšić *et al.* [7] investigated some peculiarities of electromagnetic metamaterials convenient for plasmon-based chemical sensing with enhanced sensitivity, and it was believed that metamaterial sensor may have potential applications in environmental sensing, homeland security and biosensing. Alù *et al.* [8] proposed a method of dielectric sensing using *ɛ* near-zero narrow waveguide channels. Shreiber *et al.* [9] developed a novel microwave nondestructive evaluation sensor using metamaterial lens for detection of material defects small relative to a wavelength. Zheludev [10] analysed the road ahead for metamaterials, and pointed out that sensor applications are another growth area in metamaterials research. Our team [11–13] has studied the performance of metamaterial sensors, and shown that the sensitivity and resolution of the sensors can be greatly enhanced by metamaterials.

WGM is a morphology-dependent resonance, which occurs when an electromagnetic wave travels in a dielectric medium of circular geometry. After repeated total internal reflections at the curved boundary, the electromagnetic field can close on itself and give rise to resonances [14]. The WGM resonance phenomenon has received increasing attention due to its high potential for the realization of microcavity lasers [15,16], quantum computers [17], sensing applications [18–26], *etc.* Examples of the applications of WGM sensors include biosensing [21], nanoparticle detection [22], single-molecule detection [23], temperature measurement [24], ammonia detection [25], and TNT detection [26]. However, to the best of our knowledge, there is no report about the metamaterial sensor based on microring resonator operating in WGM.

The purpose of this paper is to investigate the performance of the metamaterial microring resonator sensor and to illustrate how it is different from the traditional microring resonator sensor. We derive the dispersion relation of the hollow cylindrical dielectric waveguide, and compute the resonant frequencies and Q factors of the corresponding WGMs. We then make full wave simulation on the performance of the metamaterial sensor. This study shows that the metamaterial sensor possesses much higher sensitivity than traditional microring resonator sensor, and the mechanism behind this phenomenon is the amplification of evanescent wave.

## 2. Theoretical Analysis

**Figure 1.**
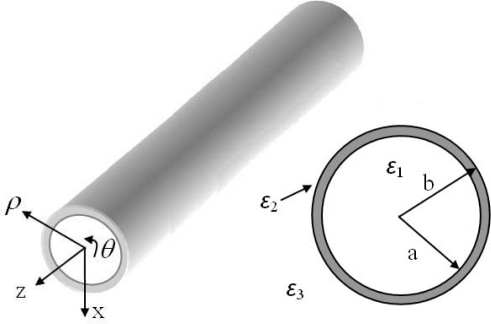
**(a)** Schematic of the cylindrical dielectric waveguide. **(b)** Cross section of the waveguide with inner radius b and outer radius a. Relative permittivity of each medium are labeled as ɛ_1_, ɛ_2_ and ɛ_3_.

The geometry of a hollow cylindrical dielectric waveguide is shown in Figure 1. A hollow core of relative permittivity *ɛ_1_* = 1 is surrounded by a concentric layer of material having relative permittivity *ɛ_2_*. The region exterior to the layer is free-space (*ɛ_3_* = 1). The axial fields in regions 1, 2, and 3 for TM mode [27] are:

(1a) $$E_{z}^{\left(1\right)}\;(r,\;\theta )\;=\;A_{m}J_{m}(p_{1}r)e^{\pm jm\theta }$$

(1b) $$E_{z}^{\left(2\right)}\;(r,\;\theta )\;=\;(B_{m}J_{m}(p_{2}r)\;+\;C_{m}Y_{m}\;(p_{2}\;r))e^{\pm jm\theta }$$

(1c) $$E_{z}^{\left(3\right)}\;(r,\;\theta )\;=\;D_{m}K_{m}\;(qr)e^{\pm jm\theta }$$

where *A_m_*, *B_m_*, *C_m_*, and *D_m_* are arbitrary constants.

The functions *J_m_*, *Y_m_*, and *K_m_* are, respectively, the Bessel functions of the first kind, of the second kind, and the modified Bessel function of the second kind. $p_{1}^{2}\;=\;\omega^{2}{ɛ}_{1}\mu_{0}\;-\;\beta^{2}\;=\;k_{1}^{2}\;-\;\beta^{2}$, $p_{2}^{2}\;=\;\omega^{2}{ɛ}_{2}\mu_{0}\;-\;\beta^{2}\;=\;k_{2}^{2}\;-\;\beta^{2}$, and $q^{2}\;=\;\beta^{2}\;-\;\omega^{2}{ɛ}_{3}\mu_{0}\;=\;\beta^{2}\;-\;k_{3}^{2}$. *k_1_* = *n_1_* *ω/c*, *k_2_* = *n_2_* *ω/c*, *k_3_* = *n_3_* *ω/c* are respectively the wave number of region 1, 2, 3, while *n_1_*, *n_2_*, *n_3_* are the corresponding reflective index. *β* is the propagation constant, and *m* is the angular order. For an infinite hollow cylindrical dielectric waveguide with negligible absorption and no axial component of the propagation constant (*β* = 0), TM mode degenerates to WGM [28], Equation (1) becomes:

(2a) $$E_{z}^{\left(1\right)}\;(r,\;\theta )\;=\;A_{m}J_{m}(k_{1}r)e^{\pm jm\theta }$$

(2b) $$E_{z}^{\left(2\right)}\;(r,\;\theta )\;=\;(B_{m}J_{m}(k_{2}r)\;+\;C_{m}Y_{m}\;(k_{2}\;r))e^{\pm jm\theta }$$

(2c) $$E_{z}^{\left(3\right)}\;(r,\;\theta )\;=\;D_{m}^{\prime }H_{m}^{\left(1\right)}\;(k_{3}r)e^{\pm jm\theta }$$

where *D′_m_* = (*iπ/2)e^im^^π^^/2^* *D_m_*, $H_{m}^{\left(1\right)}$ is the Hankel function of the first kind. The relation between $H_{m}^{\left(1\right)}$ and *K_m_* is *K_m_(−iz)* = *(iπ/2)e^im^^π^^/2^* $H_{m}^{\left(1\right)}\;(z)$.

Finally, the radial and azimuthal electric fields are derived from the axial electric field using Maxwell’s equations. For TM mode in the cylindrical dielectric waveguide, transverse magnetic fields can be obtained as:

(3a) $$H_{r}\;(r,\;\theta )\;=\;\frac{1}{p^{2}}\;\left(\frac{i\omega ɛ}{r}\cdot \frac{\partial E_{z}\;(r,\;\theta )}{\partial \theta }\right)$$

(3b) $$H_{\theta }\;(r,\;\theta )\;=\;\frac{1}{p^{2}}\left(-i\omega ɛ\cdot \frac{\partial E_{z}\;(r,\;\theta )}{\partial r}\right)$$

For WGM in the cylindrical dielectric waveguide, Equation (3) becomes:

(4a) $$H_{r}\;(r,\;\theta )\;=\;\frac{i}{Z_{0}k_{0}}\frac{1}{r}\frac{\partial }{\partial \theta }E_{z}\;(r,\;\theta )$$

(4b) $$H_{\theta }\;(r,\;\theta )\;=\;\frac{-i}{Z_{0}k_{0}}\frac{\partial }{\partial r}\;E_{z}\;(r,\;\theta )$$

The field matching equations at the boundary surface *r* = *a* and *r* = *b* are expressed as: $E_{z}^{\left(1\right)}\;(a,\;\theta )\;=\;E_{z}^{\left(2\right)}\;(a,\;\theta )$, $H_{\theta }^{\left(1\right)}\;(a,\;\theta )\;=\;H_{\theta }^{\left(2\right)}\;(a,\;\theta )$, $E_{z}^{\left(2\right)}\;(b,\;\theta )\;=\;E_{z}^{\left(3\right)}\;(b,\;\theta )$, $H_{\theta }^{\left(2\right)}\;(b,\;\theta )\;=\;H_{\theta }^{\left(3\right)}\;(b,\;\theta )$.Satisfying these conditions gives:

(5) $$[M]{\left[A_{m},\;B_{m},\;C_{m},\;D_{m}^{\prime }\right]}^{T}\;=\;0$$

where:

(6) $$[M]\;=\;\left[\begin{array}{cccc} J_{m}\;(k_{1}a) & -J_{m}\;(k_{2}a) & -Y_{m}\;(k_{2}a) & 0\\ k_{1}J_{m}^{\prime }\;(k_{1}a) & -k_{2}J_{m}^{\prime }\;(k_{2}a) & -k_{2}Y_{m}^{\prime }\;(k_{2}a) & 0\\ 0 & J_{m}\;(k_{2}b) & Y_{m}\;(k_{2}b) & -H_{m}^{\left(1\right)}\;(k_{3}b)\\ 0 & k_{2}J_{m}^{\prime }\;(k_{2}b) & k_{2}Y_{m}^{\prime }\;(k_{2}b) & -k_{3}H_{m}^{\prime (1)}\;(k_{3}b) \end{array}\right]$$

*J′_m_*, *Y′_m_*, $H_{m}^{\prime (1)}$ are the derivative of *J_m_*, *Y_m_* and $H_{m}^{\left(1\right)}$. The dispersion equation can be obtained by setting | *M* = 0|.

(7) $$\begin{array}{c} -k_{2}J_{m}\;(k_{1}a)J_{m}^{\prime }\;(k_{2}a)[-k_{3}Y_{m}\;(k_{2}b)H_{m}^{\prime (1)}\;(k_{3}b)\;+\;k_{2}H_{m}^{\left(1\right)}\;(k_{3}b)Y_{m}^{\prime }\;(k_{2}b)]\\ +k_{2}J_{m}\;(k_{1}a)Y_{m}^{\prime }\;(k_{2}a)[-k_{3}J_{m}\;(k_{2}b)H_{m}^{\prime (1)}\;(k_{3}b)\;+\;k_{2}J_{m}^{\prime }\;(k_{2}b)H_{m}^{\left(1\right)}\;(k_{3}b)]\\ +k_{1}J_{m}^{\prime }\;(k_{1}a)J_{m}\;(k_{2}a)[-k_{3}Y_{m}\;(k_{2}b)H_{m}^{\prime (1)}\;(k_{3}b)\;+\;k_{2}H_{m}^{\left(1\right)}\;(k_{3}b)Y_{m}^{\prime }\;(k_{2}b)]\\ -k_{1}J_{m}^{\prime }\;(k_{1}a)Y_{m}\;(k_{2}a)[-k_{3}J_{m}\;(k_{2}b)H_{m}^{\prime (1)}\;(k_{3}b)\;+\;k_{2}H_{m}^{\left(1\right)}\;(k_{3}b)J_{m}^{\prime }\;(k_{2}b)]\;=\;0 \end{array}$$

In the lossless case, the resonant frequencies at mode number *m* in the frequency range of 185 to 212 THz are obtained under the condition of *a* = 2.2 μm, *b* = 2.5 μm, *n*_1_ = *n*_3_ = 1, *n*_2_ = 3.2, by solving the lowest order radial WGMs of a hollow cylindrical dielectric waveguide. The results are shown in Table 1.

**Figure 2.**
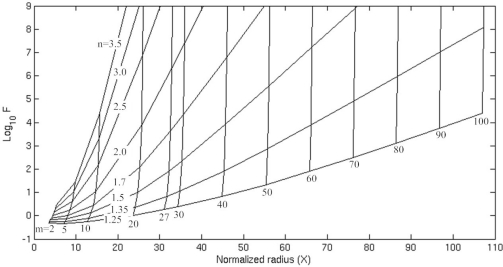
Radiation-loss-limited finesse (F) of the lowest order radial WGMs of a hollow cylindrical dielectric waveguide of index n_2_ in a medium of index n_1_ = n_3_ = 1 plotted against normalized radius (X).

In order to estimate the radiation-loss-limited Q factors of WGMs [28,29], complex frequencies of the resonances *ω* = *ω′* + *jω*″ were introduced into the dispersion equation. The real part *ω′* determines the wavelength of the resonances and the Q factor can be estimated with the expression: *Q =* *ω*′ /2*ω*″. This dimensionless parameter generalizes the imaginary part of the solution space. In order to generalize the real part, a normalized radius is defined as X = n_2_2πb/λ. To solve for the complex roots of the dispersion equation |*M*|= 0, a global optimization scheme can be used to minimize the absolute value of the equation over two variables: *Q* and X. Figure 2 displays the radiation-loss-limited finesse (F = Q/m) *vs.* normalized radius for a variety of azimuthal mode numbers and index ratios. The family of diagonal lines represents varying refractive index contrast (n = n_2_ /n_1_). The family of nearly vertical lines corresponds to WGM resonances, each characterized by an azimuthal mode number *m*. From Figure 2, we can observe that the radiation-loss-limited finesse increases with normalized radius and mode number. Besides, for a give normalized radius and mode number, the radiation-loss-limited finesse increases with the refractive index contrast. These results are beneficial for designing a microring resonator. In the following section, we will simulate the microring resonator and compare its resonant frequencies with the analytical results. Then, the metamaterial sensor constituted by the microring resonator with a layer of metamaterial loaded into its inner side and coupled to a straight waveguide is studied.

## 3. Full-Wave Simulations of the Metamaterial Sensor

### 3.1. Simulation Model

**Figure 3.**
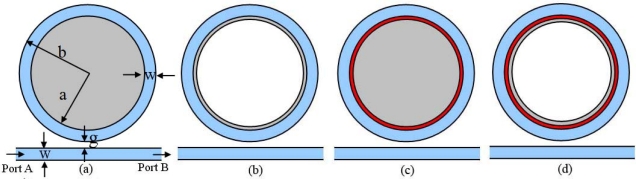
Simulation model of the microring sensor. **(a)** Conventional microring sensor with a dielectric core (colored in grey). **(b)** The dielectric sample is attached to the inner side of the microring. **(c)** A layer of metamaterial (colored in red) is added to the inner side of the microring with a dielectric core. **(d)** The dielectric sample is attached to the inner side of the microring adhere to the metamaterial layer.

Simulation models of the microring sensor are illustrated in Figure 3. Figure 3(a) shows the conventional microring sensor with a dielectric core. It is denoted as Model A in the following simulation. The width of the ring and the waveguide is w = 0.3 μm. The inner and outer radius of the ring is a = 2.2 μm and b = 2.5 μm. The distance from outer ring to the waveguide is g = 0.232 μm. The refractive index of the ring and the waveguide is n = 3.2. Figure 3(b) shows another simulation scenario, of which the dielectric sample is attached to the inner side of the microring. It is denoted as Model B. Figure 3(c) is the simulation model of the proposed metamaterial sensor that is constituted by the microring with a metamaterial layer attached to the inner side. The dielectric core is colored in grey. It is denoted as Model C. In Figure 3(d), the dielectric sample is attached to the inside of the ring adhere to the metamaterial layer. It is denoted as Model D. The permittivity and permeability of the metamaterial layer is *ɛ_r_* = *μ_r_* *=* −1. In what follows, we will simulate the performance of the traditional microring sensor and the metamaterial sensor with the help of the commercial software COMSOL multiphysics.

### 3.2. Results and Discussions

**Figure 4.**
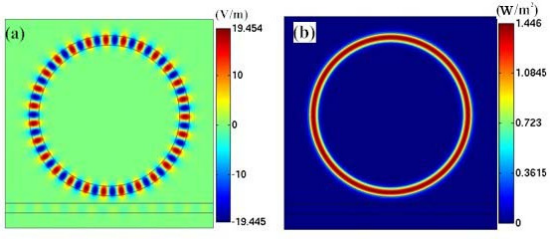

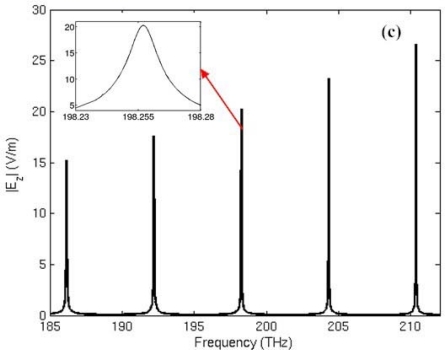
**(a)** Visualization of the steady-state electric field pattern in the air-core microring resonator at the frequency of *f_r_* = 198.257 THz (*m* = 27). **(b)** Power flow distribution. **(c)** Resonant frequency spectrum of the microring.

Firstly, the electric field distribution inside the traditional microring sensor (Model A) at the frequency of 198.257 THz is simulated, as shown in Figure 4(a). The field pattern is *m* = 27 first-order radial waveguide mode, which is formed by the circulation of electromagnetic wave within the microring. Figure 4(b) shows the power flow distribution. It is seen that at the resonant frequency, power flow is confined into the microring. In Figure 4(c), we graphed the spectrum of the microring resonator in the frequency range of 185–212 THz. It is simulated by frequency sweep. The excitation is set at port A of the waveguide.

From left to right, the spectral lines represent mode 25, 26, 27, 28 and 29 of the microring resonator. The inset portrays the amplification in the frequency range of 198.23–198.28 THz. The resonant frequency of the microring is calculated and compared with the theoretical results, as shown in Table 2. The good agreement between the simulation results (*f_s_*) and the theoretical results (*f_t_*) confirms the effectiveness of the numerical simulation.

**Figure 5.**
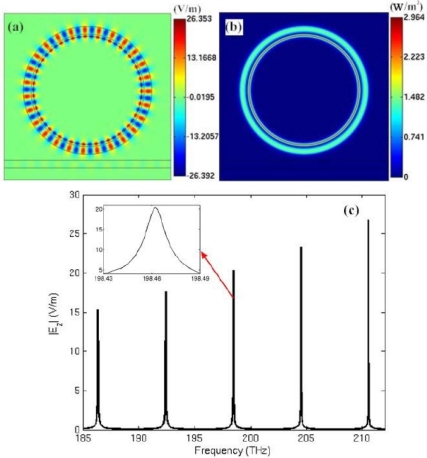
**(a)** Visualization of the steady-state electric field pattern in the metamaterial sensor at the frequency of *f_r_* = 198.46THz (*m* = 27). **(b)** The power flow distribution. **(c)** The resonant frequency spectrum.

Next, a layer of metamaterials with thickness of 0.09 μm is loaded into the inner side of an air-core microring to build the simulation model of the metamaterial sensor (Model C). Simulation results of the electric field pattern, power flow and resonant frequency spectrum of the metamaterial sensor are illustrated in panels (a), (b) and (c) of Figure 5. In this case, the maximum electric field is located in the metamaterial layer where the power flow intensity is then rather strong. A comparison between metamaterial sensor and the microring sensor is shown in Table 3. It is seen that a thin layer of metamaterials loaded into the inner side of the microring doesn’t affect the high-Q performance of the microring resonator, and has little influence on the resonant frequency. Since the maximum electric field is permeated into the metamaterial layer, this region will be very sensitive in a dielectric environment. In what follows, we investigate the performance of the metamaterial sensor in a dielectric sensor and compare it with that of the microring sensor.

**Figure 6.**
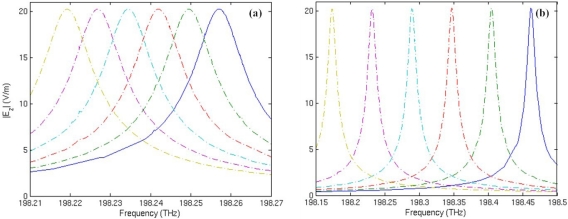
Resonant frequency spectrum of mode 27 with respect to the change of core medium permittivity *ɛ_s_*. From left to right, the curves correspond to *ɛ_s_* = 1, 1.02, 1.04, 1.06, 1.08 and 1.1, respectively. **(a)** The traditional microring sensor. **(b)** The metamaterial sensor.

Figure 6 shows the spectrum shift of mode 27 with respect to the change of core medium permittivity *ɛ_s_*. In the simulation, ɛ*_s_* is supposed to vary from 1 to 1.1 with an interval of 0.02. It is found that with the increase of the core medium permittivity, the resonant frequency always shifts to the decrescent direction. Comparing Figure 6(a) with Figure 6(b), we can clearly observe that the response to an increase of 0.02 in core medium permittivity is a frequency downshift of 7.58 GHz on average for the traditional microring sensor; for the metamaterial sensor, the average frequency shift is 57.82 GHz. Therefore, sensitivity of the metamaterial sensor is 7.6 times that of the traditional microring sensor for volume sensing.

**Figure 7.**
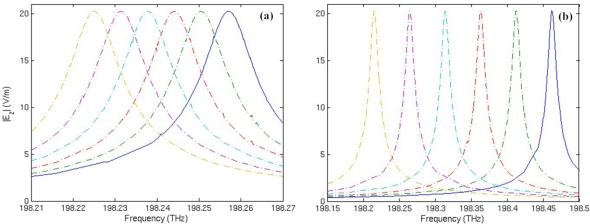
Resonant frequency spectrum of mode 27 with respect to the change of adsorbed substance permittivity *ɛ_s_*. From left to right, the curves correspond to ɛ*_s_* = 1, 1.02, 1.04, 1.06, 1.08 and 1.1, respectively. **(a)** The traditional microring sensor. **(b)** The metamaterial sensor.

Suppose that a layer of substance with thickness of t = 0.075 μm is adsorbed to the inner side of the microring (Figure 3, Model B), or adhered to the metamaterial layer (Figure 3, Model D), the spectrum shift of mode 27 with respect to the change of substance permittivity *ɛ_s_* is simulated and shown in Figure 7. Results show that the response to an increase of 0.02 in adsorbed substance permittivity is a frequency downshift of 6.35 GHz in average for the traditional microring sensor; for the metamaterial sensor, the average frequency shift is 49.34 GHz, which is about 7.8 times that of the traditional microring sensor for surface sensing.

**Figure 8.**
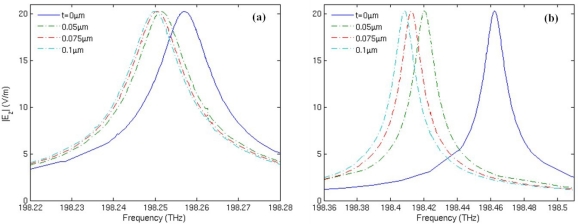
Resonant frequency spectrum of mode 27 with respect to the increase of substance layer thickness *t*. **(a)** The traditional microring sensor. **(b)** The metamaterial sensor.

Figure 8 shows the spectrum shift of mode 27 with respect to the increase of substance layer thickness *t*, which represents the adsorption or attachment of molecules. Permittivity of the substance layer is set to be 1.02 in the simulation. From Figure 8(a), we can see that when *t* increases from 0 to 0.05 μm, the frequency shift of the traditional microring sensor is 5.53 GHz; the increase of *t* from 0.05 μm to 0.1 μm with an interval of 0.025 μm results in a frequency downshift of 0.81 GHz in average. For the metamaterial sensor, a frequency shift of 42.18 GHz is induced by the increase of *t* from 0 to 0.05 μm, as shown in Figure 8(b). Besides, the increase of *t* from 0.05 μm to 0.1 μm with an interval of 0.025 μm results in a frequency downshift of 5.96 GHz in average, with is about 7.3 times that of the traditional microring sensor. Therefore, the metamaterial sensor possesses much higher sensitivity than the traditional microring sensor.

**Figure 9.**
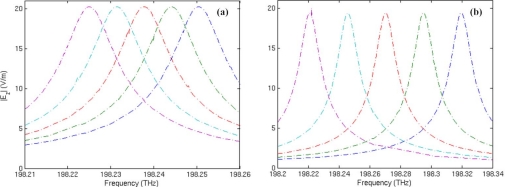

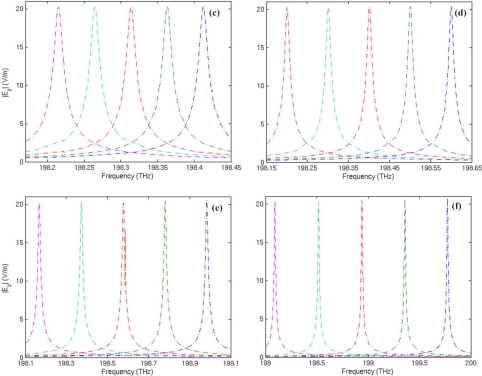
Resonant frequency spectrum of mode 27 with respect to the change of adsorbed substance permittivity ɛ*_s_*. From left to right, the curves correspond to ɛ*_s_* = 1.02, 1.04, 1.06, 1.08 and 1.1, respectively. **(a)** The traditional microring sensor. **(b–f)** show the simulation results of the metamaterial sensor with a metamaterial layer in the thickness of 0.06 μm, 0.09 μm, 0.12 μm, 0.15 μm, and 0.18 μm respectively, attached to the inner side.

To understand the impact of the metamaterials on the sensitivity of the microring sensor, we assume that the thickness (*t_m_*) of the metamaterial layer increases from 0.06 μm to 0.18 μm with an interval of 0.03 μm, and the corresponding resonant frequency spectra of mode 27 with respect to the change of adsorbed substance permittivity ɛ*_s_* are then simulated as shown in Figure 9. Thickness of the substance layer is t = 0.075 μm. The resonant frequencies *f_r_*, Q factors, and frequency shift Δ*f_r_* calculated from the data in Figure 9 are listed in Table 4. It is seen that the increase of metamaterial layer thickness will induce a larger frequency shift in dielectric sensing, but it doesn’t affect the high Q performance of the resonator.

For the traditional microring sensor (*t_m_* = 0), the response to an increase of 0.02 in adsorbed substance permittivity is a frequency downshift of 6.3 GHz on average. For the metamaterial sensor with a layer of metamaterials in the thickness of 0.06 μm, the frequency downshift is 24.5 GHz on average. When the thickness of the metamaterial layer is increased to 0.09 μm, 0.12 μm, 0.15 μm and 0.18 μm, the corresponding frequency down shift will be 49.5 GHz, 99.8 GHz, 203.7 GHz and 420 GHz on average, respectively. Therefore, sensitivity of the metamaterial sensor can be greatly improved by increasing the thickness of the metamaterial layer attached to its inner side.

**Figure 10.**
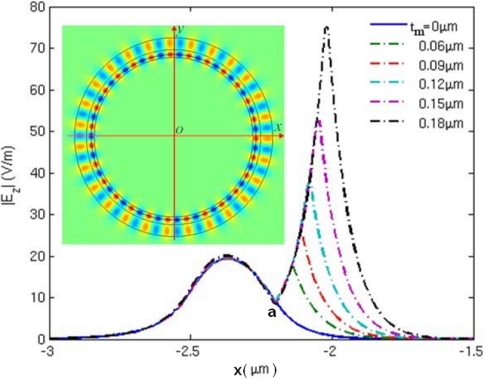
Electric field distribution along x axis from −3 μm to −1.5 μm for the metamaterial sensor operating in mode 27. The inset shows the electric field distribution of the sensor of *t_m_* = 0.12 μm.

To reveal the mechanism behind these phenomena, we plotted the electric field distribution along the x axis from −3 μm to −1.5 μm for mode 27, as shown in Figure 10. Point “a” in Figure 10 is the inner boundary of the microring resonator. When the metamaterial layer is loaded onto the inner side, inner boundary of the metamaterial sensor shifts to the right side and shown at the peak of the dashed lines in Figure 10. Permittivity and thickness of the adsorbed substance is set to be ɛ*_s_* = 1.02, t = 0.075 μm. It is seen that the electric field intensity increases with metamaterial layer thickness (*t_m_*). The inset shows the electric field distribution of the metamaterial sensor of *t_m_* = 0.12 μm. From Figure 10, we can clearly observe that the stronger electric field of evanescent wave penetrates into the detecting region when the thickness of metamaterial layer increases. Therefore, the amplification of evanescent wave is the essence for the improvement of sensitivity.

## 4 Conclusions

We have theoretically analyzed here the WGMs of a hollow cylindrical dielectric waveguide by solving the Maxwell equations with the corresponding boundary conditions. The resonant frequencies in the frequency range of 185 to 212 THz are obtained analytically, and the results reveal good agreement with full wave simulation. The metamaterial sensor constituted by a microring resonator loaded with a layer of double negative metamaterial is proposed, and considering the excitation of WGMs, its performance is simulated numerically and compared with the traditional microring sensor. We show that the metamaterial sensor possesses a higher sensitivity than the traditional microring sensor, due to the amplification of evanescent waves. Moreover, the sensitivity will be further improved by increasing the thickness of the metamaterial layer. It is expected that with the development of metamaterial technology, metamaterial sensors based on the microring resonator can be realized, and will contribute to more applications in dielectric sensing in fields where high sensitivity is required.

## Figures and Tables

**Table 1.** Mode number and resonant frequency.

*m*	*25*	*26*	*27*	*28*	*29*
*f_r_* (THz)	186.274	192.332	198.388	204.441	210.493

**Table 2.** Comparison of the theoretical resonant frequency (*f_t_*) and the simulated resonant frequency (*f_s_*) of the microring for mode 25, 26, 27, 28 and 29.

*Mode(m)*	*25*	*26*	*27*	*28*	*29*
*f_t_*(THz)	186.274	192.332	198.388	204.441	210.493
*f_s_*(THz)	186.156	192.208	198.257	204.305	210.351

**Table 3.** Simulated Q factor and resonance frequency for Model A and C.

Mode		25	26	27	28	29
Model A	Q	9279	12539	16970	22600	30145
	*f_r_*(THz)	186.156	192.208	198.257	204.305	210.351

Model C	Q	9338	12594	16952	22740	30341
	*f_r_*(THz)	186.360	192.412	198.462	204.511	210.559

**Table 4.** Q factor, resonant frequency and frequency shift calculated from Figure 9.

*ɛ_s_*		*1.02*	*1.04*	*1.06*	*1.08*	*1.1*
*t_m_(μm)*						
0	Q	16896	16914	16870	16868	16955
	*f_r_*(THz)	198.250	198.244	198.238	198.231	198.225
	Δ*f_r_*(GHz)	6.0	6.0	7.0	6.0	
0.06	Q	15502	15503	15502	15501	16513
	*f_r_*(THz)	198.320	198.295	198.270	198.246	198.222
	Δ*f_r_*(GHz)	25.0	25.0	24.0	24.0	
0.09	Q	16758	16885	16901	16890	16841
	*f_r_*(THz)	198.413	198.363	198.314	198.265	198.215
	Δ*f_r_*(GHz)	50.0	49.0	49.0	50.0	
0.12	Q	17006	17049	16932	16915	16867
	*f_r_*(THz)	198.600	198.501	198.401	198.301	198.201
	Δ*f_r_*(GHz)	99.0	100.0	100.0	100.0	
0.15	Q	17151	17067	17022	16914	16830
	*f_r_*(THz)	198.983	198.781	198.577	198.373	198.168
	Δ*f_r_*(GHz)	202.0	204.0	204.0	205.0	
0.18	Q	17470	17393	17129	16948	16746
	*f_r_*(THz)	199.772	199.357	198.939	198.517	198.092
	Δ*f_r_*(GHz)	415.0	418.0	422.0	425.0	
